# Retraction Note: HnRNP A1 - mediated alternative splicing of CCDC50 contributes to cancer progression of clear cell renal cell carcinoma via ZNF395

**DOI:** 10.1186/s13046-023-02613-4

**Published:** 2023-02-08

**Authors:** Guoliang Sun, Hui Zhou, Ke Chen, Jin Zeng, Yangjun Zhang, Libin Yan, Weimin Yao, Junhui Hu, Tao Wang, Jinchun Xing, Kefeng Xiao, Lily Wu, Zhangqun Ye, Hua Xu

**Affiliations:** 1 Department of Urology, Tongji Hospital, Tongji Medical College, Huazhong University of Science and Technology, Wuhan, 430030 P.R. China; 2 Hubei Institute of Urology, Wuhan, 430030 P.R. China; 3 Department of Molecular and Medical Pharmacology, David Geffen School of Medicine, University of California at Los Angeles, Los Angeles, CA 90095 USA; 4 Department of Urology, The First Affiliated Hospital of Xiamen University, Xiamen, 361000 P.R. China; 5 Department of Urology, The People’s Hospital of Shenzhen City, Shenzhen, 518000 P.R. China

**Retraction Note: J Exp Clin Cancer Res 39, 116 (2020)**

**https://doi.org/10.1186/s13046-020-01606-x**

The Editor-in-Chief has retracted this article because of the following concerns:

- Within Figure 3D, the panels (OS-RC-2 vs sh-CCDC50#1) and (OS-RC-2 vs sh-CCDC50#2) overlap.
- Figure 3H contained a panel (CCDC50-S vs whole) with missing sections within the image.
- An incorrect panel (OS-RC-2 vs sh-HnRNP A1+CCDC50-FL) was provided for supplementary figure 5B.

The data reported in this article are therefore unreliable.

Guoliang Sun and Hua Xu agree to this retraction. Hui Zhou has agreed to this retraction but not to the wording of this retraction notice. Ke Chen, Jin Zeng, Yangjun Zhang, Libin Yan, Weimin Yao, Junhui Hu, Tao Wang, Jinchun Xing, Kefeng Xiao, Lily Wu and Zhangqun Ye have not responded to any correspondence from the publisher about this retraction.

